# Occurrence of Natural and Synthetic Micro-Fibers in the Mediterranean Sea: A Review

**DOI:** 10.3390/toxics10070391

**Published:** 2022-07-13

**Authors:** Saul Santini, Eleonora De Beni, Tania Martellini, Chiara Sarti, Demetrio Randazzo, Roberto Ciraolo, Costanza Scopetani, Alessandra Cincinelli

**Affiliations:** 1 Department of Chemistry “Ugo Schiff”, University of Florence, Via della Lastruccia 3, Sesto Fiorentino, 50019 Florence, Italy; saul.santini@unifi.it (S.S.); eleonoradebeni@msn.com (E.D.B.); chiara.sarti@unifi.it (C.S.); demetrio.randazzo@unifi.it (D.R.); roberto.ciraolo@unifi.it (R.C.); 2 Department of Chemistry “Ugo Schiff”, University of Florence and Consorzio Interuniversitario per lo Sviluppo dei Sistemi a Grande Interfase (CSGI), Via della Lastruccia 3, Sesto Fiorentino, 50019 Florence, Italy; 3 Faculty of Biological and Environmental Sciences Ecosystems and Environment Research Programme, University of Helsinki, Niemenkatu 73, FI-15140 Lahti, Finland; costanza.scopetani@helsinki.fi

**Keywords:** microplastics, fibers, cellulose, Mediterranean Sea, pollution, chemical characterization, environmental pollution, biota contamination

## Abstract

Among microplastics (MPs), fibers are one of the most abundant shapes encountered in the aquatic environment. Growing attention is being focused on this typology of particles since they are considered an important form of marine contamination. Information about microfibers distribution in the Mediterranean Sea is still limited and the increasing evidence of the high amount of fibers in the aquatic environment should lead to a different classification from MPs which, by definition, are composed only of synthetic materials and not natural. In the past, cellulosic fibers (natural and regenerated) have been likely included in the synthetic realm by hundreds of studies, inflating “micro-plastic” counts in both environmental matrices and organisms. Comparisons are often hampered because many of the available studies have explicitly excluded the micro-fibers (MFs) content due, for example, to methodological problems. Considering the abundance of micro-fibers in the environment, a chemical composition analysis is fundamental for toxicological assessments. Overall, the results of this review work provide the basis to monitor and mitigate the impacts of microfiber pollution on the sea ecosystems in the Mediterranean Sea, which can be used to investigate other basins of the world for future risk assessment.

## 1. Introduction

**Figure 1:**
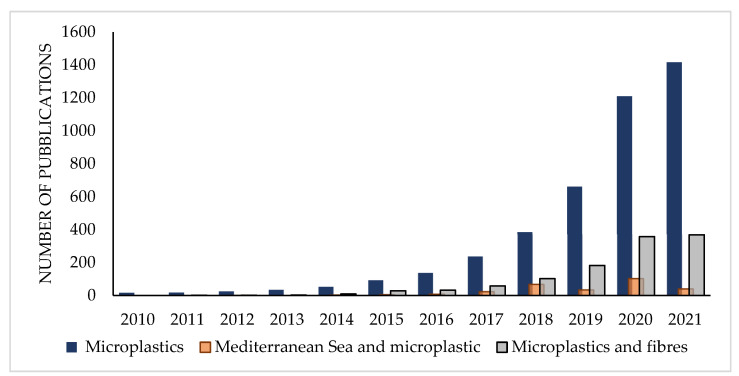
Number of publications per year studying MPs in the environment, MPs in the Mediterranean Sea and MPs/fibers. Source: Web of Science Database.

Plastic is considered a persistent and ubiquitous pollutant, and it is considered among the top environmental concerns of the Anthropocene [1,2]. Microplastics (MPs) are small plastic fragments ranging from 1 μm to 5 mm in size that can be found in different environmental compartments [3]. MPs accumulate in the environment and increase stress on the marine, freshwater and terrestrial ecosystems [4]. Several studies have evidenced their presence in the marine environment [5,6,7], aquatic sediments [8], freshwaters [9], soils [10] and the atmosphere [11,12]. MPs can act as a carrier of hydrophobic organic contaminants, transporting the pollutants inside the organisms through ingestion and subsequent chemical release. However, it has been shown that sometimes, ingested MPs can adsorb the pollutants already present in the organisms and remove them once they are excreted [13]. Plastics themselves contain toxic chemical additives (such as plasticizers, antistatic agents, flame retardants, heat stabilizers, acid scavengers, colorants, etc.) that can be released into the environment [14]. Moreover, chemical additives in plastics can adsorb organic contaminants from other matrices and increase the exposure of several contaminants to the environment [15,16]. These chemicals, if present in the food chain and absorbed by humans, could cause many diseases linked with hormonal disruption, reproductive problems, nervous tissue, liver and kidney damage, etc. [17]. Although the effects of plastic litter on the marine environment and organisms have been recently investigated in several oceanic areas, more information is needed for the Mediterranean Sea [18], which is an enclosed sea with limited exchange with the ocean basins and high diversity of sensitive ecosystems. This particularity, together with other factors such as the high-density population in the coastal areas, intense navigation traffic, and industrial and fishing activities, makes the Mediterranean basin one of the most affected seas by plastic accumulation all over the world [19]. The determination and characterization of MPs for shape, color, size and type is fundamental to better understand their impact on the environment. Among MPs, fibers are the predominant shape in the aquatic environment, often accounting for more than 80% of the total items [11,20,21,22,23,24,25,26,27,28,29,30,31,32,33]. For this reason, increasing attention is being paid to micro-fibers and their potential toxicological and environmental effects, as evidenced by the growing number of studies on microfiber pollution over the past decade (Figure 1). According to the general definition proposed by Liu et al. (2019), microfibers (MFs) are any natural or artificial fibrous materials of threadlike structure with a diameter less than 50 μm, length ranging from 1 μm to 5 mm, and length to diameter ratio greater than 100 [34]. 

Microplastics, especially MFs, contaminate and affect many aquatic organisms or species of birds or mammals that feed on aquatic species since they are often mistaken for food and ingested by prey species, which, in turn, are eaten by predators, allowing MPs to move up the trophic chain [35,36]. 

However, information about the microfiber distribution in the Mediterranean Sea is still limited and filling this knowledge gap would be the first step to take to tackle the microfiber pollution issue. The second important step is to characterize the nature of the fibers because they are not always plastic but rather dyed cellulose. In the last decade, cellulosic fibers (natural and regenerated) have been likely included in the synthetic realm by hundreds of studies, inflating “microplastic” counts in both environmental matrices and organisms; this error has resulted from the assumption that all colored fibers are synthetic [37]. The separation of textile MFs from other MPs does not necessarily add complexity but, conversely, might bring consistency to the comparison across different investigations [38]. A recent study by Pedrotti et al., 2021, shows that fibers analyzed from textiles considered 100% synthetic constituted 17.4% of natural or derived from the transformation of natural polymers. In the seawater samples, 14–50% of the fibers analyzed were synthetic, 35–72% were of natural origin (cotton, wool) or made by processing natural polymers (especially cellulose), and the rest were a mixture of different materials or could not be identified (14–21%) [39]. Most microfibers of natural origin come from anthropogenic sources; however, a very small percentage can be released into the environment from “natural” sources such as bast fibers, leaf fibers, seed fibers, grass and all other types such as roots and wood [40]. As shown in a study by Athey et al., 2021, many of the methods used to investigate the occurrence of MPs do not provide data on the nature of synthetic or non-synthetic. Moreover, some steps of the methods, such as chemical digestion, could generate mistakes [41,42,43,44]. Comparisons between different studies are often hampered because many of them highlight the predominance of fibers in environmental samples without including a chemical characterization of the fibers. Thus, to ensure that studies of the presence of microplastics in the environment, and particularly in the marine environment, provide information to understand the ecological damage from these pollutants, it is essential to use appropriate instrumentation. While a stereomicroscope is sufficient to separate MFs from MPs, more complex instrumentation is required to identify the nature of the MPs and specifically whether an MF is natural or synthetic, cellulose or not. To this aim, chemical analysis of the polymeric composition using, for example, Fourier Transform Infrared Spectroscopy (FTIR), µ-Raman and scanning electron microscope (SEM) [5] need to be performed. The present review aims at examining the current literature on the occurrence of cellulose and cellulose-based fibers in the Mediterranean Sea, providing a picture of MF contamination in coastal marine environments.

### Non-Synthetic MFs Toxicity

In the industry of non-synthetic textiles, a similar cocktail of dyes and chemicals as in synthetic textiles is used, and many of these substances are toxic and can accumulate in the environment [45]. The toxic chemicals released by MPs into the tissue of fishes and marine animals are several and include, e.g.**,** colorants, plasticizers, elasticizers, and together with the microfiber particles, can physically damage various organs, the digestive tract, stomach lining, immune function and stymie growth, and thus, affect the entire ecosystem [44,46]. The textile industry, a source of pollution of MPs, including MFs, in the environment, involves the use of many dyes that can be toxic to organisms [47,48]. Several dyes such as: Acid Red 26, Basic Red 9, Basic Violet 14, Direct Black38, Direct Blue 6, Direct Red 28, Disperse Blue 1, Disperse Orange11 and Disperse Yellow 3 are classified as carcinogenic in the European standard of textile ecology [49]. The effect of the carcinogenic dyes in rats is included in the IARC monographs [50]. Moreover, experiments were conducted to observe the toxic effects of these dyes if dispersed in the environment and absorbed by marine organisms. Shen et al. (2015) studied the toxic effects of Basic Violet 14, Direct Red 28 and Acid Red 26 on zebrafish larvae, observing acute effects: cardiovascular toxicity and molecular mechanism by Acid Red 26 and hepatotoxicity effects by Basic Violet 14 [51]. In a study by Remy et al. (2015), the presence of non-synthetic fibers was identified in the invertebrate community that live in Neptune grass, Posidonia Oceanica (L.) Delile, a species heavily predated by fishes, in the Mediterranean coastal zone [25]. The dyes of these fibers were two: Direct Red 28 and Direct Blue 22, and they are used in the textile industry for natural and artificial fibers. Direct Blue 22 is not considered harmful to humans, but Direct Red 28 is classified as carcinogenic, mutagenic or toxic to reproduction. Direct Red 28 can be reduced by the intestine bacteria and generate carcinogenic molecules in humans [52]. Non-synthetic and semi-synthetic microfibers and their additives or dyes may interact negatively with biota in aquatic environments similar to plastic microfibers, but ingestion, chemical leaching and degradation rates in marine environments are poorly understood [25]. Natural fibers, although considered environmentally friendly by their faster environmental degradation, pose a global threat comparable to synthetic polymers. In fact, due to the processing of textiles, they can be mixed with flame retardants and/or resins, and this not only represents a problem related to the release of toxic compounds but also has an effect on degradation times, which become longer [37]. Moreover, since they constitute a major component of litter in water bodies and aquatic animals, they could become important vectors not only of contaminants but also of bacteria [53]. Espinosa et al. (2016) have associated the presence of MFs in fish with a mixture of several polybrominated diphenyl ethers at concentrations that can cause effects on the endocrine system [54]. The presence of these substances in the environment can hamper reproduction, in particular, for fish. This is due to the high sensitivity of juvenile and adult fishes to endocrine disruptors [55,56]. The adverse effects caused to the aforementioned organisms by fibers might be relevant also for humans since MPs and their associated chemicals can be transferred through the food chain and reach us [57]. Another way through which the human organism is exposed to MPs is airborne contamination. The MPs get deposited in our lung tissues and lead to lung inflammation [58]. These fibers are known to have adverse impacts on terrestrial and marine ecosystems [59]. Unfortunately, MPs are present in all environmental compartments and rayon, and polyester fibers are commonly present in marine animal species [60]; they can be absorbed through herds and cause problems to the respiratory and gastrointestinal systems. The aim of this review is to report the current state of research on the environmental impacts of microfibers and to identify gaps in knowledge. In light of the findings, it appears essential that future research should focus on the characterization of microfibers, the chemical and physical properties of various fabrics, both synthetic and natural, and the ability of microfibers to become carriers of toxic substances.

## 2. Discussion

We summarize the 2015–2021 literature data on the abundance of fibers in the Mediterranean Sea, including the abundance of synthetic or non-synthetic fibers, colors and size. Based on published literature from the Web of Science, SCOPUS, Google Scholar, Science Direct, Pubmed and Sci-Finder, we obtained studies by searching for “microfibers and microplastics”, “microplastics and fibers”, “filaments and plastic pollution”, “plastic and microfibers”, “microplastics and filaments”, “microplastic fibers”, “synthetic fibers and microplastics”, “Textile fibers and microplastics”, “fragments and microfibers”, Microplastics and Mediterranean sea”, Microplastics and biota”, Sediment and microplastics”, “Microfibers and source and fate”, “Microfibers and toxic effects”. Then, we eliminated irrelevant studies by reading the title and abstract and supplemented our literature database by reading all references of the selected papers. Moreover, only available data on fiber abundance in the Mediterranean Sea over the 2015–2021 timeframe for biota, sediment and seawater were selected, and they are summarized in Table 1 and Table 2, respectively. Finally, we selected 49 studies. 

### 2.1. Most Abundance Shapes 

The available literature data on the abundance of fibers in the Mediterranean Sea in the time frame 2015–2021 for biota, sediment and seawater are summarized in Table 1 and Table 2, respectively.

**Figure 2:**
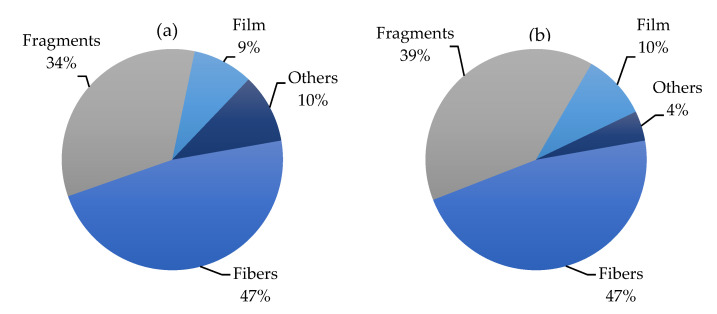
Pie charts showing the relative abundance (%) of fibers, fragments, films and other shapes (i.e., spheres, pellets, sheets) in the literature data globally in biota (**a**) and water (**b**) from the Mediterranean Sea.

Figure 2 summarizes all data presented in Table 1 and Table 2, providing a global view of the occurrence of fibers, fragments, films and other shapes (i.e.**,** spheres, pellets, sheets) in the Mediterranean Sea. The uniformly high presence of MFs in the water environment and biota samples of the Mediterranean area reflect a wider distribution of sources of textile fibers along the coastlines of the Mediterranean Sea, but also, the potential for atmospheric transport is much higher for MFs than for MPs [38]. 

In the Mediterranean Sea, MFs account for approximately 40% (range 1.6–85.9%) of fragments of micrometric size in the seawater and seabed, followed by fragments (mean 34.5%, range 1.6–72.7%), films (mean 17.3%, range 1.5–14.1%) and other shapes, such as spheres, pellets and sheets (mean 8.2%, range 1.6–24.1%). When considering MPs occurrence in marine organisms (invertebrates, fishes and sea turtles) collected from the Mediterranean Sea, we found 39.1% fragments, 37.8% fibers, 14.5% films and 8.7% other shapes. The matrices containing the higher amounts of fibers were sediments and seawater, where they reached 43.9%. The remaining part was formed by fragments (26.8%), films (22%) and others (7.3%).

Microfiber pollution has also been documented in all major ocean basins [21,22,23,28,37,98] as well as within the entire trophic web [20,24,29,32,33,59,99,100,101,102,103,104]. Natural microfibers are infrequently documented and not typically included in marine environment impact analyses, resulting in the underestimation of a potentially ubiquitous and harmful pollutant [28]. The literature data on the abundance of non-synthetic materials, including natural (i.e.**,** cellulose), artificial (i.e.**,** cellulose-based) and other (i.e.**,** wool, silk and natural rubber) MFs, found in the Mediterranean Sea, are shown in Table 3 and Table 4 for biota and sediment and seawater samples, respectively. The number of investigated individuals, the total amount of fibers, and the sub-sample analyzed are also reported. Table 3 focuses on the literature data on the abundance of natural (i.e.**,** cellulose), artificial (i.e.**,** cellulose-based), other non-synthetic (i.e.**,** wool, silk) and plastic microfibers in biota (invertebrates, fish and sea turtles) of the Mediterranean Sea, together with the number of specimens sampled and the relative number of fibers found and analyzed. 

### 2.2. Non-Synthetic Composition of MFs in the Mediterranean Sea

Studies are increasingly documenting the ingestion of cellulose fibers by fishes and other organisms. A large portion of MFs found in biota from the Mediterranean Sea is cellulose-based, which consists of both dyed natural cellulose and manufactured fibers composed of regenerated cellulose. Natural fibers originating from plants are grouped into seed (e.g., cotton), bast (e.g., flax, hemp, kenaf, ramie), leaf (e.g., sisal) as well as tree fibers (e.g.**,** wood), which have been extensively used for clothing, domestic woven fabrics and ropes for thousands of years [105]. Over the last years, and due to their wide availability, low cost, good recyclability, low density and high-specific mechanical strength, natural fibers have aroused interest in several applications as reinforcements in, e.g., the automotive and construction industries [106]. Wood pulp is the most important resource for producing cellulose-based human-made fibers, which can be manufactured through derivative and direct methods [107]. Human-made cellulosic materials represent a good compromise as the fiber-forming processes currently in use can lead to innovative fiber materials that combine the advantages of natural fibers and the possibility of tailor-made properties and chemical modifications [108]. In Europe, fibers and fabrics produced from regenerated cellulose are known as “viscose” whereas in the U.S., they are called rayon. Rayon makes up a significant proportion of synthetic microparticles found in the marine environment [20]. Rayon is used in cigarette filters, personal hygiene products and clothing and is introduced to the marine environment through sewage (e.g.**,** washing of clothes) [23]. 

As reported above, Remy et al. (2015) identified the presence of artificial fibers in invertebrate communities; the artificial fibers were made of viscose, and the chemical characterization was confirmed by Raman spectroscopy. In addition, the colors of these fibers were two: Direct Red 28 and Ingrain Blue. These colors are used in the textile industry both for natural and artificial fibers. This shows that specific dyes cannot be linked to natural only or artificial only fibers, and thus, dyes cannot be used as reliable indicators for identifying synthetic or natural MFs or MPs [25]. 

Similar levels of non-synthetic fibers were detected in sea cucumbers, *Holothuria Tubulosa* (Gmelin, 1788), from Croatia, in which cellulose and cellulose acetate in stomach contents reached 13.3% and 14.8% in samples collected from Silba Island and Telašćica, respectively (ranging within 0–33.3% of total items). In the same study proposed by Renzi and Blašković (2020), fibers represented the larger number of recorded MPs in sediments from both Silba and Telašćica (ranging within 0–67.9% of total items). Among benthic species, sea cucumbers were selected as a target because they are widely representative of marine benthic species and are considered a key benthic taxonomic group to preserve marine ecosystem integrity (they are listed as protected species in some EU countries). Moreover, they play a crucial role in the food web through predation by stars, crustaceans, gastropods and fishes [77]. The presence of anthropogenic fibers both in *H. Tubulosa* and sediments (see Table 3) shows the large diffusion of these pollutants, supporting the hypothesis of active ingestion by these organisms from the surrounding environment. Similar results were obtained from Boskovic et al., 2021, where cellulose fibers in nine out of ten sediment samples of the Montenegrin coast were detected, which highlighted the predominance of fibers among all other MPs [96]. PP was detected in all the different sampling locations, while PE was in seven out of ten. The results showed the highest concentrations of MPs were in locations near highly populated centers, municipal effluent discharge restaurants, fishing and tourist activities, such as cruises.

The semipelagic fish bogue *Boops boops* (Linnaeus, 1758) is a commonly agreed-upon bioindicator in the Mediterranean Sea [18]. Italy is one of the European countries required to implement the Marine Strategy Framework Directive (MSFD), and the use of bioindicator species is strongly recommended by MSFD and other monitoring programs (e.g.**,** UNEP/MAP) to increase the knowledge on the extent of marine litter pollution and its impacts on marine species [109]. Since *B. boops* is an omnivorous species, which feeds both benthic and pelagic preys, living on diverse types of the sea bottom (sandy, muddy, rocky and seagrass beds) [100], it has been proposed to act as a sentinel for microplastic pollution in the Mediterranean small-scale pelagic environment (https://plasticbustersmpas.interreg-med.eu, accessed on 15 June 2022). In the study conducted by Savoca et al. (2019) in the Gulf of Patti [72], the authors reported, for the first time in the Mediterranean Sea, the ingestion of human-made cellulose fibers in bogue specimens, assuming that the high presence of fibers found in their stomach might depend on the habitat and its extension. As a matter of fact, the urban wastewater treatment of the area is not powerful enough to retain all the fibers, especially during the summer when many tourists populate the area [110]. Their data complied with the studies of Fastelli et al. (2016) and Cannas et al. (2017) carried out in the same area of the Mediterranean Sea [86,89]. Similar results were also obtained by Rios-Fuster et al. (2019), who evaluated the ingestion of anthropogenic particles in four species of fish, including *B. boops* [30], and found a percentage of 92.86% of fibers and 7.14% of fragments. Previous studies carried out using the same species as a bioindicator detected similar MFs occurrence levels in the Balearic Islands [100]. In this study, a total of 731 items were observed in 195 full gastrointestinal tracts of bogue. The fibers were only detected and characterized by different colors. Similarly, Neves et al. (2015) recorded a total of 73 MPs in the 32 bogues sampled in the North Atlantic, off the Portuguese coast, 48 of which (65.8%) were fibers and 25 (34.2%) were particles [24]. On the contrary, Garcia-Garin et al. (2019) found a prevalence of fragments (60%) in bogues samples collected from the Spanish Catalan coast near Barcelona. The authors suggested that the high amount of fragments found in the organisms was due to the severe MPs pollution present in the sampling area [71].

Avio et al. (2020) provided a comprehensive characterization of the ingestion of microplastics in several fish and invertebrate species from the Adriatic Sea, which is considered a preferential area of plastic accumulation in the Mediterranean. Almost 500 organisms, including benthic and pelagic invertebrates and benthopelagic, pelagic and demersal fish species, were collected (see Table 3). Textile MFs were abundant in Adriatic food webs occurring in all the analyzed species with frequencies (ranging between 40% and 70%) higher than those reported for MPs; an elevated percentage of MFs was of natural (74% cotton, 8% wool) and non-synthetic origin (8%) [38]. One of the species studied by Avio et al. (2020) was the European hake, *Merluccius Merluccius* (Linnaeus, 1758), which is an important predatory species inhabiting a wide range of depths (20–1000 m) throughout the Mediterranean Sea and the north-eastern Atlantic region. It is one of the main commercial and most exploited species of fish in all northern Mediterranean countries [111]. Bellas et al. (2016) and Giani et al. (2019) investigated the occurrence of MPs in *M. Merluccius,* and their results were comparable to those of Avio et al. (2020): the detected MPs were mostly constituted by fibers (71% and 81%, respectively). In both studies, however, no chemical characterization of the fibers was provided [26,73]. Interestingly, previous studies conducted by Suaria et al. (2015) and Avio et al. (2015) in the same area of the Mediterranean Sea reported a predominance of fragments over fibers in plankton and *M. Merluccius* specimens (78.5% and 57%, respectively). 

The usual hake diet consists mainly of Crustacea (especially Decapoda) and teleost fishes (i.e., *Engraulis Encrasicolus* and *Cepola microphthalmia*). European anchovy *E. Encrasicolus*, together with *Sardina pilchardus* (Walbaum, 1792), are some of the most captured fish species in the Mediterranean Sea and are thus of economic importance. Moreover, they are directly subjected to MP’s pollution because they are planktivorous and are mainly filter-feeding. Both of the species have been used in MP studies, and natural and plastic microparticles have been found in both of the organisms with a predominance of MFs (83%) [61,62]. Natural fibers (such as cotton) accounted for 54.1% and other cellulose-based fibers for 12.5%. Plastic materials, especially PET, PE and PA, accounted for 33.3%. A study conducted by Collard et al. (2015) showed that the majority of “non-plastic” particles found in *E. Encrasicolus* collected from the Gulf of Lions were made of cellulose (54.3%) [67]. Similar results to those presented by Collard et al. (2015) and Compa et al. (2018) in the same Mediterranean area were confirmed by Sanchez-Vidal et al. (2018) [67]. Sanchez-Vidal et al. (2018) reported the predominance of cellulosic fibers (79.7%) over other synthetic polymers (see Table 4) in the sediment on the Spanish Mediterranean coast [92]. Moreover, a recent study carried out in the Southern Tyrrhenian Sea by Savoca et al. (2020) confirmed the presence of polymers, such as PP, PA, Nylon and PE, and human-made cellulose, such as rayon, in *E. Encrasicolus*, and *S. Pilchardus*. Instead, Neves et al. (2015) noted the presence of MPs in fish from the coast of Portugal, highlighting the presence of rayon fibers through μ-FTIR, one of the techniques more suitable for distinguishing and determining the chemical composition of fibers [24].

Red mullet, *Mullus Barbatus* (Linnaeus, 1758), and striped red mullet, *Mullus surmuletus* (Linnaeus, 1758), are demersal fish species widely spread in the Mediterranean Sea and the NE Atlantic [78], and are considered important resources for coastal Mediterranean fisheries [112]. Due to its dietary habits, *M. Barbatus* is in constant contact with sediment and, therefore, it is exposed to the pollutants present in this matrix. Thus, it has been widely proposed as a sentinel species for several pollutants. Fiber ingestion by the red mullet has been widely reported in *M. Barbatus* samples collected from several areas of the Mediterranean Sea, including the Turkish shore, Adriatic and Tyrrhenian Seas and the Mediterranean Spanish Coast [26,27,61,73,75,78]. It is interesting to note that some of these studies showed that 56.79% of the fibers found in the fishes were cellulose-based, almost twice as many as PET (31.14%) [78]. 

*M. Surmuletus* is sensitive to marine debris contamination and microplastic ingestion [112]. In the study carried out by Alomar et al. (2017), the vast majority of identified microplastics in *M. Surmuletus* samples were filaments (30% of which were non-plastic material) [64]. 

Capillo et al. (2020) investigated five demersal fish species from the Southern Tyrrhenian Sea, including the red mullet *M. Barbatus*, the piper gurnard *Trigla Lyra* (Linnaeus, 1758) and the blackmouth catshark *Galeus Melastomus* (Rafinesque, 1810). A total of 97.1% of the microparticles found in all the samples were fibers. Specifically, the red mullet presented high values of plastic material (mainly PTFE, 75%), while the items found in specimens of *T. Lyra* were all composed of cellulose (100%). The feeding behavior of *T. Lyra* is the same of *M. Barbatus*, i.e.**,** the fish swallows sediment (together with the prey) and then expels them through the gills. 

*G. Melastomus* has a different feeding behavior compared to *T. Lyra*; it is a benthopelagic predator that feeds mainly on demersal invertebrates (shrimps and cephalopods) and mesopelagic fish. It could ingest MPs during predation, biomagnifying them along the food chain. The estimation of the percentage of MFs ingestion in *G. Melastomus* in this study (especially nylon) is different from those reported in other areas of the Mediterranean Sea [75]. Indeed, a high proportion of cellulosic-based fibers in this species was found in samples from the Balearic Islands (western Mediterranean Sea) area, where Alomar and Deudero (2016) reported the dominance of cellophane over other synthetic polymers. In the stomachs of this elasmobranch species, the authors showed that 86.36% of the identified particles were filaments, while the rests were fragments and films. Woodall et al. (2014), Sanchez-Vidal et al. (2018), Filgueiras et al. (2019) and Suaria et al. (2020) suggested that *G. Melastomus* ingests fibers directly from the seafloor and water column [19,22,37,65,92]. Similar results were achieved by Valente et al. (2019), who identified the presence of 221 synthetic fibers (85.7% of the particles) in *G. Melastomus* collected from the Tyrrhenian Sea. These data comply with the results reported in a study conducted by Cannas et al. (2017) in the same part of the Mediterranean [74,89]. Anastasopoulou et al. (2013) have also recorded MP ingestion by *G. Melastomus* in the Ionian Sea, but unlike the results obtained from the previous studies, the percentage of fibers reached only 3% [113]. In agreement with these results, Ruiz-Orejòn et al. (2016) reported 87.3% of hard plastic fragments as the majority of the material observed in the Ionian Sea, demonstrating how the marine environment can affect biota microparticles ingestion [87]. 

Finally, in a recent study by Sayed et al. (2021) along Egypt’s coast, the presence of cellulose-based fibers was observed while analyzing the level of MPs in the digestive tracts of *Caranx Crysos*, *Liza Aurata*, *Siganus Rivulatus* and *Epinephelus Caninus* from the Eastern Harbor. Plastic particles were evident in all fish samples, including seven thermoplastic polymers. Rayon and polyethylene terephthalate were the most dominant types of polymers in fish [83].

Due to the concentrations of plastic in the Mediterranean Sea, loggerhead sea turtles, *Caretta Caretta* (Linneaus, 1758), were confirmed by Matiddi et al. (2017) as the main target species for monitoring MP ingestion by marine organisms. The turtles tend to ingest marine litter, confusing it with natural prey [114]. The study conducted by Duncan et al. (2018) provides an overview of the presence of microplastics in various marine turtle specimens. The analysis of marine turtles’ specimens reveals a high abundance of fibers unanimous in the three basins (Atlantic 77.1%, Mediterranean Sea 85.3%, Pacific 64.8%). Of these, a subsample of the isolated particles was tested using FTIR to determine the polymeric composition, revealing the presence of both synthetics (mainly PE, ethylene propylene, PEST and polyacrylamide) and cellulose-based materials (rayon, natural rubber and plant protein) [69].

### 2.3. Color of MFs

**Figure 3:**
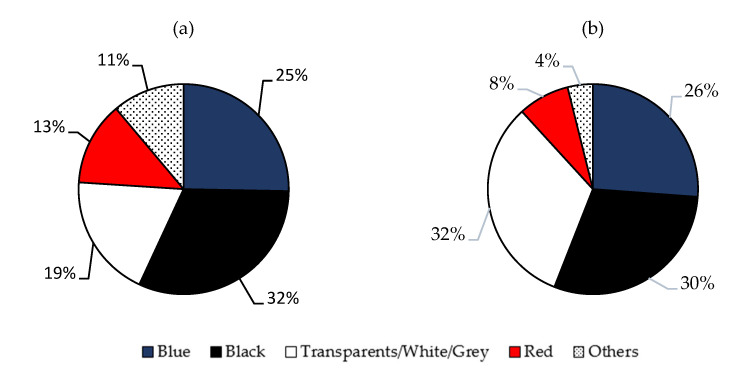
Most abundant colors in MFs present in the literature data from the Mediterranean Sea, both in the biota (**a**), and in seabed and seawater samples (**b**).

From the available literature data, four different colors in MFs were found to be more abundant in the Mediterranean Sea, both in biota (fishes, invertebrates and sea turtles) and in the seabed and seawater samples. As indicated in Figure 3, the dominant color was black (ranged between 12.1–100%), followed by transparent and clear colors (2.5–50.3%) and blue (10.1–45.8%). Red (3.8–27%) and others (2.2–20%) were less abundant. 

Instead, in open basins (Atlantic, Pacific, Indian Arctic and Southern Oceans) the following order was observed: blue (10.1–88%) > black (8.8–57.1%) > transparent (2.5–47%) > red (5.2–42%) > others (1–9%). The MF’s color could potentially increase their bioavailability due to their resemblance to prey objects. There is evidence of visual confusion between prey and anthropogenic particles [30]. Predatory fish show a preference for ingesting blue fibers, while transparent fibers may be confused due to their resemblance to gelatinous prey or can be ingested accidentally via filtration [76]. Furthermore, studies noticed, without providing any explanation, that planktivorous fish seem to ingest whiter, lighter and bluer fiber colors [115]. The only speculation that was made to explain this observation was that these colors are the most abundant found in the fibers collected from the Mediterranean Sea. Another aspect that has to be taken into account is that some chemical treatments used during the extraction procedure of the fibers can cause physical damage and discoloration of the microplastics, as shown by Cole et al. (2014) [116].

### 2.4. Self-Contamination

During the analysis of MFs, one of the biggest problems is the contamination of the sample by those who carry out the sampling, treatment and analysis of the samples. Contaminations can occur through the use of instrumentation that releases particles into the environment or from the researcher’s clothing [117]. The procedures attempt to control contaminants entering samples from analyst clothing, airborne sources, laboratory surfaces, equipment and consumables used, but there is not yet a standardized method to prevent contaminations. Over the years, more and more precautions have been taken for the treatments of the samples, in fact, initially, the procedures did not take into account the possible self-contamination [118], while techniques have recently been adopted to avoid this problem [119,120,121]. For example, Gaylarde et al. (2021) cleaned all materials used with ethanol and filtered deionized water, put on colored suits and performed the fish dissection and digestion protocols in a clean airflow cabin [122]; instead, Barrows et al. (2018) tested microplastic contamination during the treatment of the sample: cleaning all laboratory surfaces, analyzing laboratory water and laboratory air and analyzing blanks of the filtrate used to rinse the sample bottle and filtration apparatus. The results showed average contamination of 0.005 pieces per 0.010 L of water and 0.154 pieces per 8 min of exposure to air from synthetic and non-synthetic MPs [28]. As highlighted by Prata et al. (2021), less than 50% of studies on MPs do not collect and analyze controls and blanks during the sampling phase and processing step of the sample [119]. Moreover, only some studies involve taking “control” samples of possible sources of contamination from MPs and MFs and the use of colored cotton clothes [96,121,122,123,124,125]). Finally, as highlighted in a study by Scopetani et al. (2020), the level of self-contamination in MPs studies is not negligible, highlighting the importance of finding a standardized method to avoid the overestimation of MPs and MFs in environmental matrices [126].

### 2.5. Size 

**Figure 4:**
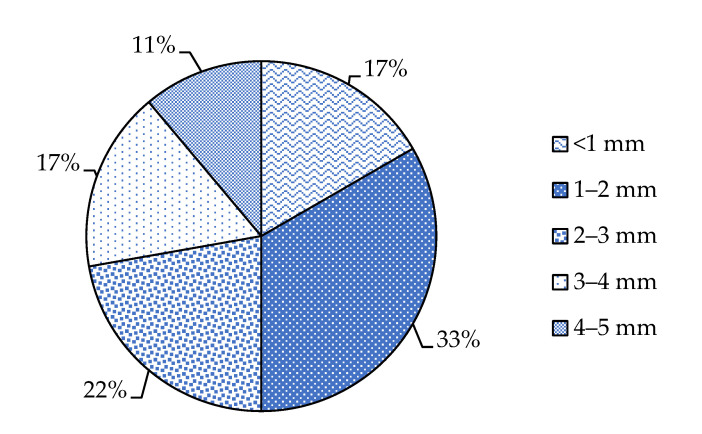
A comparison of the literature data of percentages frequency of different fiber lengths in biota and water samples from the Mediterranean Sea.

From the data available in the literature, we can notice that most of the studies conducted in the Mediterranean Sea that focused on microfibers pollution investigated microfibers with a length ranging between 1 and 2 mm (Figure 4). 

We can hypothesize that this may probably represent the optimal size to carry out investigations regarding the chemical composition of the fibers, but further research is needed to deepen this aspect. The small size of the MFs is relevant as it determines the potential impact of these contaminants on the ecosystem and the bioaccumulation/biomagnification in biota from ingestion. If the fibers are ingested by marine organisms, they can damage them, block and affect the physical performance of the digestive tract of fish [20]. The effect caused by the volume occupied in the digestive tracts does not depend on the size of the individual fibers because these can tangle and form larger agglomerates. Indeed, fibers longer than 5 mm (usually not considered in studies on microplastics) can tangle with themselves and with other fibers and occupy large volumes in the stomach, volumes similar to those of agglomerates of shorter fibers [20,80]. Therefore, it is difficult to find a correlation between fiber size and the effects on the organism, but if they do not tangle, as shown in a study by Grigorakis et al. (2017), they can cross the entire digestive tract and be expelled from the body without causing damage [127]. Not all the studies agree on the possibility of detecting the presence of microplastics up to 0.6 mm in organs not belonging to the digestive system, as detected by Avio et al. (2015) in fish mullet liver [62]. Instead, many authors believe that probably only MPs and MFs smaller than 100 µm or their additives can come into contact with organs not belonging to the digestive system and cross the intestinal barrier [78].

## 3. Conclusions

As described above, the investigations in the Mediterranean Sea provide insight into the level of microfiber pollution and underline the necessity to use specific analytical techniques to explore and confirm MFs composition to avoid overestimation when assessing the level of MP occurrence in the marine environment. This review underlines the need to distinguish natural fibers from plastic ones, given the high number of fibers found in the marine environment and biota. Additionally, future studies should better investigate the impact of fibers on biota since synthetic fibers tangle easily and can originate bundles of fibers causing obstruction in organs and hindering or preventing feeding. The same consideration is applied to cellulosic fibers, even if they do not constitute an environmental problem in themselves, but any additives or dyes within them could potentially be carcinogenic and harmful to sea organisms and, consequently, to humans. Overall, the results of this review provide the basis to monitor the impacts of microfiber pollution on the sea ecosystems in the Mediterranean Sea, which can be used to investigate other basins of the world for future risk frameworks.

## Figures and Tables

**Table 1:** Literature review about percentages of the predominant type of microplastic (fibers, fragments) in the Mediterranean Sea, region and year of sampling and instrumental method for the characterization of MPs in biota (invertebrates, fishes and sea turtles).

Area	Year of Sampling	Predominant Type (%)	Instrumental Method	References
Calvi Bay (Corsica)	2011–2012	All fibrous in shape	Raman	[25]
Southern Adriatic Sea	2013	78.5% fragments	FTIR	[61]
Central and North Adriatic Sea	2014	57% fragments	FTIR	[62]
Gulf of Lions (France)	2013	37.1%. fibers	Raman	[63]
Spanish Mediterranean coast	2014	71% fibers	n.a.	[26]
Mediterranean coast of Turkey	2015	70% fibers	FTIR	[27]
Mallorca Island (Balearic Islands, Western Mediterranean)	2014–2015	97% fibers	FTIR	[64]
Mallorca Island (Balearic Islands, Western Mediterranean)	n.a.	86.4% fibers	FTIR	[65]
Giglio Island	2014	60% fragments	FTIR	[66]
Western Spanish Mediterranean coast	2015	83% fibers	FTIR	[67]
Northern Ionian Sea(*M. galloprovincialis; S. pilchardus, P. erithrinus, M. barbatus*)	2015	77.8% fragments	FTIR	[68]
		80% fragments		
		73.3% fragments		
		83.3% fragments		
Northern Cyprus	n.a.	85.3% fibers	FTIR	[69]
Adriatic and NE Ionian Sea (Croatian Sea; Slovenian Sea, NE Ionian sea)	2014–2015	75.6% fibers	n.a.	[70]
		97.7% fibers		
		79% fragments		
Spanish Catalan coast	2018	~60% fragments	FTIR	[71]
Tyrrhenian Sea (Northern coasts of Sicily, Gulf of Patti)	2019	93.3% fibers	µ-Raman, XPS and SEM-EDX	[72]
Mediterranean Sea (European hake, Red mullet)	n.a.	81% fibers	n.a.	[73]
		44% fibers		
Anzio coast (south of Rome, Tyrrhenian Sea)	2018	85.7% fibers	FTIR	[74]
Tyrrhenian Sea (northern coasts of Sicily, Gulf of Patti	2017	97.1% fibers	ATR-FTIR and µ-Raman	[75]
Iberian Peninsula coast and Balearic Islands (Western Mediterranean Sea)	2015	92.9% fibers	n.a.	[30]
Ligurian Sea	2011–2014	n.a. fibers	FTIR	[76]
Silba Island and Telašćica (Croatia, Adriatic Sea)	2007 and 2018	39.4–43.3% fibers	µ-FTIR	[77]
		35.7–57.5% fibers		
NW Mediterranean (Catalan coast)	n.a.	97% fibers	Raman	[78]
Northern, Central and Southern Adriatic Sea (Pelagic, benthopelagic, demerdal and benthic organism)	2016	38% fragments	µ-FTIR	[38]
		50% fragments		
		53% fragments		
		61% fragments		
Gulf of Patti (Southern Tyrrhenian Sea)	2019	93.3% fibers	FTIR and Raman	[79]
Catalan coast (NW Mediterranean Sea)	2007, 2017 and 2018	84.6% fibers	FTIR	[80]
Southeast Spain	2018, 2019	71.7% fibers	FTIR	[81]
Turkey, Izmir bay	2020	87.2% fibers	n.a.	[82]
Egypt cost (Mars Mtruh, Port Said, Alexandria, Damietta)	2020	100% fibers	ATR-FTIR	[83]
		50% fragments		
		96.2% fragments		
		85.2% fragments		

n.a: not available.

**Table 2:** Literature review about percentages of the predominant type of microplastic (fibers, fragments) in the Mediterranean Sea, region and year of sampling and instrumental method for characterization of MPs in sediments and seawaters.

Area	Year of Sampling	Predominant Type (%)	Instrumental Method	References
Mediterranean Sea	2001–2012	All fibrous in shape	FTIR	[22]
Gulf of Lion, the BalearicIslands, Sardinia and Corsica	2012	72% fragments	n.a.	[84]
Southern Adriatic Sea	2013	78.5% fragments	FTIR	[61]
Mediterranean Sea	2013	n.a.	ATR-FTIR	[85]
Aeolian Archipelago (central Mediterranean and Tyrrhenian sea)	n.a.	>85% fibers	n.a.	[86]
Mediterranean cost of Turkey	2015	70% fibers	FTIR	[27]
Balearic Islands, Adriatic and Ionian Sea	2011 and 2013	87.3% fragments	n.a.	[87]
Israeli Mediterranean coast	2013–2015	96.2% fragments	n.a.	[88]
Tyrrhenian Sea	2012	>88% fibers	n.a.	[89]
Central Adriatic Sea	2015	69.3% fibers	FTIR	[90]
Northern Tunisian coast(South Lake of Tunis, North Lake of Tunis, Carthage, Goulette)	2017	66.8% fibers	FTIR	[91]
		87.3% fibers		
		71% fibers		
		98.8% fibers		
Alboran, Catalan, Cretan and Levantine Sea	2009–2015	All fibrous in shape	FTIR	[92]
Ebro River Delta (Catalonia, Spain, Northwestern Mediterranean) (Sand, benthic sediment, surface water)	2017	89.5% fibers	µ-FTIR	[93]
		75.1% fibers		
		46.1% fibers		
Spanish Mediterranean Coast	2014–2015	82.9% fibers	n.a.	[19]
Silba Island and Telašćica (Croatia, Adriatic Sea)	2007 and 2018	33.1–76.9% fibers	µ-FTIR	[77]
		82.7–97.3% fibers		
Central-western Mediterranean Sea	2017	All fibrous in shape	FTIR	[94]
Mediterranean Sea	2017	All fibrous in shape	µ-FTIR	[37]
Northwestern Mediterranean Sea (Naples, Corsica north and south-east cost of France)	2014	All fibrous in shape	FTIR	[39]
Danube delta	2018	74.6% fibers	ATR-FTIR	[95]
Montenegrin cost	2019	55.5% fibers	ATR-FTIR	[96]
Ligurian Sea coastal	2018	80% fibers	n.a.	[97]

**Table 3:** The literature data on the abundance of natural (i.e., cellulose), artificial (i.e., cellulose-based), other non-synthetic (i.e., wool, silk) and plastic microfibers in biota (invertebrates, fish and sea turtles) of the Mediterranean Sea, together with the number of specimens sampled and the relative number of fibers found and analyzed. Polyvinyl chloride (PVC), polyethylene terephthalate (PET), polypropylene (PP), polyethylene (PE), polyamide (PA), polyester (PEST), polystyrene (PS).

Species	No of Individuals	The total Amount of MFs	No of Identified MFs	Plastic Materials (%)									Non-Synthetic Materials (%)			Reference
				PVC	PET	PP	PE	PA	Nylon	PEST	PS	Others	Cellulose	Cellulose-Based	Others	
Macroinvertebrates *	235	91	11											100		[25]
*Holothuria tubulosa* (Gmelin, 1788) (Telaščica, Silba)	170	n.a.	n.a.	13.2		21.6	27.5	1.2	9.8			13.9	12.7			[77] **
				17.6	12.9	12.8	12.7		11.6		5.3	25.8	1.4			
Invertebrates and fishes *	<500	2079	100					1		10			74	8	7	[38]
*Boobs boops* (Linnaeus, 1758)	30	80	16										n.a.			[72]
Teleosts *	125	18	n.a.			31.2	6.2		12.5			31.2		18.7		[75] **
Elasmobranchs *																
*Mullus barbatus* (Linnaeus, 1758)	118	167	39		31.1								56.8			[78]
*Mullus surmuletus* (Linnaeus, 1758)	417	n.a.	n.a.		36.3							33.3		30.3		[64]
*Galeus melastomus* (Rafinesque, 1810)	125	n.a.	n.a.		27.3	12.1	4.5	3				19.7		33.3		[65]
*Caretta caretta* (Linnaeus, 1758)	102	811	169				20.7	4.9				61.2		5.8	7.4	[69]
*Chelonia mydas* (Linnaeus, 1758)																
*Engraulis encrasicolus* (Linnaeus, 1758)	9	35	19		45.7								54.3			[63] **
Plankton	29	1140	n.a.			10	41	3		12	5	22	7			[61] **
*Sardina pilchardus* (Walbaum, 1792)	105	41	24		12.5		8.3	4.2				8.3	54.1	8.3	4.2	[67] **
*Engraulis encrasicolus* (Linnaeus, 1758)																
*Sardina pilchardus* (Walbaum, 1758)	264	n.a.	n.a.			n.a.	n.a.	n.a.						n.a.		[79] **
*Engraulis encrasicolus* (Linnaeus, 1758)																
Sparus aurata (Linnaeus, 1758)	17	279	n.a.	2.2		2.9	21.5	2.2		4.4	2.2	71.3				[81]
Caranx crysos (Mitchill, 1815), Liza aurata (Risso, 1810), Siganus rivulatus (Rüppell, 1828), and Epinephelus caninus (Valenciennes, 1843)	3	480	n.a.		35.0		6.7		5			16.6		36.7		[83]
	3	383	n.a.		23.3				6.7			15.7		53.3		
	3	526	n.a.		18.8		8.4		8.4			6.7		56.7		
	3	648	n.a.		18.8		8.4		8.4			6.7		56.7		

n.a. not available. * Macroinvertebrates: *Gammarella fucicola* (Leach, 1814), *Gammarus aequicauda* (Martynov, 1931), *Melita hergensis* (Reid, 1939), *Nototropis guttatus* (Costa, 1853), *Nebalia strausi* (Risso, 1826), *Palaemon xiphias* (Risso, 1816), *Liocarcinus navigator* (Herbst, 1794), *Athanas nitescens* (Leach, 1813), *Galathea intermedia* (Liljeborg, 1851); invertebrates: *Mytilus galloprovincialis* (Lamarck, 1819), *Ostrea edulis* (Linnaeus, 1758), *Sabella spallanzanii* (Gmelin, 1805), *Actinia* sp., *Squilla mantis* (Linnaeus, 1758), *Penaeus kerathuru*s (Forskål, 1775), *Nephrops norvegicus* (Linnaeus, 1758), *Palaemon* sp., *Paracentrotus lividus* (Lamarck, 1816), *Mnemiopsis leydi* (Agassiz, 1865), *Rhizostoma pulmo* (Macri, 1778); fishes: *Sardina pilchardus* (Walbaum, 1792), *Scomber scombrus* (Linnaeus, 1758), *Trachurus trachurus* (Linnaeus, 1758), *Merluccius merluccius* (Linnaeus, 1758), *Mullus barbatus* (Linnaeus, 1758), *Chelidonichthys lucernus* (Linnaeus, 1758), *Solea solea* (Linnaeus, 1758), *Sardinella aurita* (Valenciennes, 1847), *Diplodus vulgaris* (Geoffroy Saint-Hilaire, 1817), *Pagellus erythrinus* (Linnaeus, 1758), *Spondilosoma cantharus* (Linnaeus, 1758), *Tracinus draco* (Linnaeus, 1758), *Lithognathu mormyrus* (Linnaeus, 1758); *Teleosts: M. barbatus and Trigla lyra* (Linnaeus, 1758); *Elasmobranchs: Galeus melastomus* (Rafinesque, 1810), *Scyliorhinus canicula* (Linnaeus, 1758) and *Raja miraletus* (Linnaeus, 1758); *Sparus aurata* (Linnaeus, 1758); *Caranx crysos* (Mitchill, 1815), *Liza aurata* (Risso, 1810), *Siganus rivulatus* (Rüppell, 1828) and *Epinephelus caninus* (Valenciennes, 1843). ** in this study, percentages refer not only to fibers composition but to MPs generally.

**Table 4:** The literature data on the abundance of natural (i.e.**,** cellulose), artificial (i.e.**,** cellulose-based), other non-synthetic (i.e.**,** wool, silk) and plastic microfibers in the sediment and water column from the Mediterranean Sea, together with the number of specimens sampled and the relative number of fibers found and analyzed.

Sample	No of Samples	The Total Amount of MFs	Subset of MFs for Analysis	Plastic Materials (%)											Non-Synthetic Materials (%)			Reference
				PVC	PET	PP	PE	PA	Nylon	PEST	PAN	PS	Others	Cellulose		Cellulose-Based	Others	
Sediment	12	n.a.	n.a.				23	14.7					5.3			56.9		[22]
	29	202	all		12.9	1	1	1					4.5	79.7				[92]
Sediment (Telaščica)	51	n.a.	n.a.	23					26.9				22.6	9.7		3.6		[77] *
Sediment(Silba)				18	16.2	14	12.2		17.2			1.2	6.8	13.7		0.8		
Beaches	5	197	25			8	16	24		12			16	12				[93] *
Sediment	n.a.	229																
Surface water	n.a.	293																
Seawater	29	1140	n.a.			10	41	3		12		5	22	7				[61] *
	916	23,593	2134			0.4		0.3	0.7	6.2			0.7	79.5			12.3	[37] **
	108	5466	336			0.9		0.6	0.9	4.2			0.3	47.3		39.6	5.4	[94]
Seawater (Haliotis outfall)	9	65	27			9		30		22	13	17		35				[39] ***
Seawater (Point B)	9	23	15					17		33	17		33	72				
Seawater (Bastia)	9	32	24					60		40				58				
Seawater (Dyfamed)	9	178	38					38		62				47				
Surface water	12	3289	93			33.3	30.1	1.1		1.1	4.3	4.3	3.3					[95]
Sediment	10	688	103			54.5	9.7	2.0					22.2	5.1			6.4	[96]

n.a. not available. * percentages refer not only to fiber composition but to MPs generally. ** percentages refer not only to the Mediterranean Sea but also include ocean basins. *** plastic material percentages refer only to 14–50% of synthetic material and the non-synthetic material percentages to total microfibers.

## References

[1] Abeynayaka A., Kojima F., Miwa Y., Ito N., Nihei Y., Fukunaga Y., Yashima Y., Itsubo N. (2020). Rapid Sampling of Suspended and Floating Microplastics in Challenging Riverine and Coastal Water Environments in Japan. Water.

[2] Cozzolino L., Nicastro K.R., Zardi G.I., de los Santos C.B. (2020). Species-specific plastic accumulation in the sediment and canopy of coastal vegetated habitats. Sci. Total Environ..

[3] Hartmann N.B., Hüffer T., Thompson R.C., Hassellöv M., Verschoor A., Daugaard A.E., Rist S., Karlsson T., Brennholt N., Cole M. (2019). Are We Speaking the Same Language? Recommendations for a Definition and Categorization Framework for Plastic Debris. Environ. Sci. Technol..

[4] Antunes J., Frias J., Sobral P. (2018). Microplastics on the Portuguese coast. Mar. Pollut. Bull..

[5] Cincinelli A., Martellini T., Guerranti C., Scopetani C., Chelazzi D., Giarrizzo T. (2019). A potpourri of microplastics in the sea surface and water column of the Mediterranean Sea. TrAC Trends Anal. Chem..

[6] Cincinelli A., Scopetani C., Chelazzi D., Lombardini E., Martellini T., Katsoyiannis A., Fossi M.C., Corsolini S. (2017). Microplastic in the surface waters of the Ross Sea (Antarctica): Occurrence, distribution and characterization by FTIR. Chemosphere.

[7] Wu P., Huang J., Zheng Y., Yang Y., Zhang Y., He F., Chen H., Quan G., Yan J., Li T. (2019). Environmental occurrences, fate, and impacts of microplastics. Ecotoxicol. Environ. Saf..

[8] Hurley R., Woodward J., Rothwell J.J. (2018). Microplastic contamination of river beds significantly reduced by catchment-wide flooding. Nat. Geosci..

[9] Klein S., Worch E., Knepper T.P. (2015). Occurrence and spatial distribution of microplastics in river shore sediments of the rhine-main area in Germany. Environ. Sci. Technol..

[10] Nizzetto L., Bussi G., Futter M.N., Butterfield D., Whitehead P.G. (2016). A theoretical assessment of microplastic transport in river catchments and their retention by soils and river sediments. Environ. Sci. Process. Impacts.

[11] Dris R., Gasperi J., Rocher V., Tassin B. (2018). Synthetic and non-synthetic anthropogenic fibers in a river under the impact of Paris Megacity: Sampling methodological aspects and flux estimations. Sci. Total Environ..

[12] Gasperi J., Wright S.L., Dris R., Collard F., Mandin C., Guerrouache M., Langlois V., Kelly F.J., Tassin B. (2018). Microplastics in air: Are we breathing it in?. Curr. Opin. Environ. Sci. Health.

[13] Koelmans A.A., Bakir A., Burton G.A., Janssen C.R. (2016). Microplastic as a Vector for Chemicals in the Aquatic Environment: Critical Review and Model-Supported Reinterpretation of Empirical Studies. Environ. Sci. Technol..

[14] Hahladakis J.N., Velis C.A., Weber R., Iacovidou E., Purnell P. (2018). An overview of chemical additives present in plastics: Migration, release, fate and environmental impact during their use, disposal and recycling. J. Hazard. Mater..

[15] Wagner M., Scherer C., Alvarez-Muñoz D., Brennholt N., Bourrain X., Buchinger S., Fries E., Grosbois C., Klasmeier J., Marti T. (2014). Microplastics in freshwater ecosystems: What we know and what we need to know. Environ. Sci. Eur..

[16] Kärrman A., Schönlau C., Engwall M. (2016). Exposure and Effects of Microplastics on Wildlife. A review of existing data. DiVA.

[17] Anbumani S., Kakkar P. (1999). Ecotoxicological effects of microplastics on biota: A review. Environ. Sci. Pollut. Res..

[18] Fossi M.C., Pedà C., Compa M., Tsangaris C., Alomar C., Claro F., Ioakeimidis C., Galgani F., Hema T., Deudero S. (2018). Bioindicators for monitoring marine litter ingestion and its impacts on Mediterranean biodiversity. Environ. Pollut..

[19] Filgueiras A.V., Gago J., Campillo J.A., León V.M. (2019). Microplastic distribution in surface sediments along the Spanish Mediterranean continental shelf. Environ. Sci. Pollut. Res..

[20] Lusher A.L., McHugh M., Thompson R.C. (2013). Occurrence of microplastics in the gastrointestinal tract of pelagic and demersal fish from the English Channel. Mar. Pollut. Bull..

[21] Lusher A.L., Tirelli V., O’Connor I., Officer R. (2015). Microplastics in Arctic polar waters: The first reported values of particles in surface and sub-surface samples. Sci. Rep..

[22] Woodall L.C., Sanchez-Vidal A., Canals M., Paterson G.L.J., Coppock R., Sleight V., Calafat A., Rogers A.D., Narayanaswamy B.E., Thompson R.C. (2014). The deep sea is a major sink for microplastic debris. R. Soc. Open Sci..

[23] Obbard R.W., Sadri S., Wong Y.Q., Khitun A.A., Baker I., Richard C. (2014). Who Where Why—Wordpress blog—Community mapping examples. Earth’s Future.

[24] Neves D., Sobral P., Ferreira J.L., Pereira T. (2015). Ingestion of microplastics by commercial fish off the Portuguese coast. Mar. Pollut. Bull..

[25] Remy F., Collard F., Gilbert B., Compère P., Eppe G., Lepoint G. (2015). When Microplastic Is Not Plastic: The Ingestion of Artificial Cellulose Fibers by Macrofauna Living in Seagrass Macrophytodetritus. Environ. Sci. Technol..

[26] Bellas J., Martínez-Armental J., Martínez-Cámara A., Besada V., Martínez-Gómez C. (2016). Ingestion of microplastics by demersal fish from the Spanish Atlantic and Mediterranean coasts. Mar. Pollut. Bull..

[27] Güven O., Gökdağ K., Jovanović B., Kıdeyş A.E. (2017). Microplastic litter composition of the Turkish territorial waters of the Mediterranean Sea, and its occurrence in the gastrointestinal tract of fish. Environ. Pollut..

[28] Barrows A.P.W., Cathey S.E., Petersen C.W. (2018). Marine environment microfiber contamination: Global patterns and the diversity of microparticle origins. Environ. Pollut..

[29] Bessa F., Barría P., Neto J.M., Frias J.P.G.L., Otero V., Sobral P., Marques J.C. (2018). Occurrence of microplastics in commercial fish from a natural estuarine environment. Mar. Pollut. Bull..

[30] Rios-Fuster B., Alomar C., Compa M., Guijarro B., Deudero S. (2019). Anthropogenic particles ingestion in fish species from two areas of the western Mediterranean Sea. Mar. Pollut. Bull..

[31] Hossain M.S., Rahman M.S., Uddin M.N., Sharifuzzaman S.M., Chowdhury S.R., Sarker S., Nawaz Chowdhury M.S. (2020). Microplastic contamination in Penaeid shrimp from the Northern Bay of Bengal. Chemosphere.

[32] Parton K.J., Godley B.J., Santillo D., Tausif M., Omeyer L.C.M., Galloway T.S. (2020). Investigating the presence of microplastics in demersal sharks of the North-East Atlantic. Sci. Rep..

[33] Iliff S.M., Wilczek E.R., Harris R.J., Bouldin R., Stoner E.W. (2020). Evidence of microplastics from benthic jellyfish (Cassiopea xamachana) in Florida estuaries. Mar. Pollut. Bull..

[34] Liu J., Yang Y., Ding J., Zhu B., Gao W. (2019). Microfibers: A preliminary discussion on their definition and sources. Environ. Sci. Pollut. Res..

[35] Bal B., Ghosh S., Das A.P. (2018). Microbial recovery and recycling of manganese waste and their future application: A review. Geomicrobiol. J..

[36] Mohanty S., Ghosh S., Bal B., Prasad A. (2018). A review of biotechnology processes applied for manganese recovery from wastes. Rev. Environ. Sci. Bio/Technol..

[37] Suaria G., Achtypi A., Perold V., Lee J.R., Pierucci A., Bornman T.G., Aliani S., Ryan P.G. (2020). Microfibers in oceanic surface waters: A global characterization. Sci. Adv..

[38] Avio C.G., Pittura L., d’Errico G., Abel S., Amorello S., Marino G., Gorbi S., Regoli F. (2020). Distribution and characterization of microplastic particles and textile microfibers in Adriatic food webs: General insights for biomonitoring strategies. Environ. Pollut..

[39] Pedrotti M.L., Petit S., Eyheraguibel B., Kerros M.E., Elineau A., Ghiglione J.F., Loret J.F., Rostan A., Gorsky G. (2021). Pollution by anthropogenic microfibers in North-West Mediterranean Sea and efficiency of microfiber removal by a wastewater treatment plant. Sci. Total Environ..

[40] Karimah A., Ridho M.R., Munawar S.S., Adi D.S., Ismadi, Damayanti R., Subiyanto B., Fatriasari W., Fudholi A. (2021). A review on natural fibers for development of eco-friendly bio-composite: Characteristics, and utilizations. J. Mater. Res. Technol..

[41] Cai H., Du F., Li L., Li B., Li J., Shi H. (2019). A practical approach based on FT-IR spectroscopy for identi fi cation of semi-synthetic and natural celluloses in microplastic investigation. Sci. Total Environ..

[42] Conley K., Clum A., Deepe J., Lane H., Beckingham B. (2019). Wastewater treatment plants as a source of microplastics to an urban estuary: Removal efficiencies and loading per capita over one year. Water Res. X.

[43] Helcoski R., Yonkos L.T., Sanchez A., Baldwin A.H. (2019). Wetland soil microplastics are negatively related to vegetation cover and stem density. Environ. Pollut..

[44] Athey S.N., Erdle L.M. (2021). Are We Underestimating Anthropogenic Micro fi ber Pollution? A Critical Review of Occurrence, Methods, and Reporting. Environ. Toxicol. Chem..

[45] O’neill C., Hawkes F.R., Hawkes D.L., Lourenço N.D., Pinheiro H.M., Delée W. (1999). Colour in textile effluents-sources, measurement, discharge consents and simulation: A review. J. Chem. Technol. Biotechnol..

[46] Kwak J.I., Liu H., Wang D., Lee Y.H., Lee J.S., An Y.J. (2022). Critical review of environmental impacts of microfibers in different environmental matrices. Comp. Biochem. Physiol. Part C Toxicol. Pharmacol..

[47] Verma Y. (2008). Acute toxicity assessment of textile dyes and textile and dye industrial effluents using Daphnia magna bioassay. Toxic Ind. Health.

[48] Ferraz E.R., Li Z., Boubriak O., de Oliveira D.P. (2012). De Current Issues Hepatotoxicity Assessment of the Azo Dyes Disperse Orange 1 (DO1), Disperse Red 1 (DR1,) and Disperse Red 13 (DR13) in HEPG2 Cells. J. Toxicol. Environ. Health Part A.

[49] OEKO-TEX OEKO-TEX. https://www.oeko-tex.com/en/.

[50] No C.A.S. (2016). Agents Classified by the IARC Monographs. Lancet Oncol..

[51] Shen B., Liu H., Ou W., Eilers G., Zhou S. (2015). Toxicity induced by Basic Violet 14, Direct Red 28 and Acid Red 26 in zebrafish larvae. J. Appl. Toxicol..

[52] IARC (2012). Chemical agents and related occupations. IARC Monographs on the Evaluation of Carcinogenic Risks to Humans.

[53] McCormick A.R., Hoellein T.J., London M.G., Hittie J., Scott J.W., Kelly J.J. (2016). Microplastic in surface waters of urban rivers: Concentration, sources, and associated bacterial assemblages. Ecosphere.

[54] Espinosa C., Esteban M.Á., Cuesta A., Microplastics in Aquatic Environments and and Their Toxicological Implications for Fish Licens. InTech 2016, 113–145..

[55] Zhao Y., Wang C., Xia S., Jiang J., Hu R., Yuan G., Hu J. (2014). Biosensor medaka for monitoring intersex caused by estrogenic chemicals. Environ. Sci. Technol..

[56] Rochman C.M., Lewison R.L., Eriksen M., Allen H., Cook A.M., Teh S.J. (2014). Polybrominated diphenyl ethers (PBDEs) in fish tissue may be an indicator of plastic contamination in marine habitats. Sci. Total Environ..

[57] Meeker J.D., Sathyanarayana S., Swan S.H. (2009). Phthalates and other additives in plastics: Human exposure and associated health outcomes. Philos. Trans. R. Soc. B Biol. Sci..

[58] Das A., Mishra S. (2010). Biodegradation of the metallic carcinogen hexavalent chromium Cr(VI) by an indigenously isolated bacterial strain. J. Carcinog..

[59] Taylor M.L., Gwinnett C., Robinson L.F., Woodall L.C. (2016). Plastic microfibre ingestion by deep-sea organisms. Sci. Rep..

[60] Bergmann M., Gutow L., Klages M. (2015). Marine Anthropogenic Litter.

[61] Suaria G., Avio C.G., Lattin G., Regoli F., Aliani S., Marche A.I. (2015). Neustonic microplastics in the Southern Adriatic Sea. Prelim. Results Micro.

[62] Avio C.G., Gorbi S., Regoli F. (2015). Experimental development of a new protocol for extraction and characterization of microplastics in fish tissues: First observations in commercial species from Adriatic Sea. Mar. Environ. Res..

[63] Collard F., Gilbert B., Eppe G., Parmentier E., Das K. (2015). Detection of Anthropogenic Particles in Fish Stomachs: An Isolation Method Adapted to Identification by Raman Spectroscopy. Arch. Environ. Contam. Toxicol..

[64] Alomar C., Sureda A., Capó X., Guijarro B., Tejada S., Deudero S. (2017). Microplastic ingestion by Mullus surmuletus Linnaeus, 1758 fish and its potential for causing oxidative stress. Environ. Res..

[65] Alomar C., Deudero S. (2017). Evidence of microplastic ingestion in the shark Galeus melastomus Rafinesque, 1810 in the continental shelf off the western Mediterranean Sea. Environ. Pollut..

[66] Avio C.G., Cardelli L.R., Gorbi S., Pellegrini D., Regoli F. (2017). Microplastics pollution after the removal of the Costa Concordia wreck: First evidences from a biomonitoring case study. Environ. Pollut..

[67] Compa M., Ventero A., Iglesias M., Deudero S. (2018). Ingestion of microplastics and natural fibres in Sardina pilchardus (Walbaum, 1792) and Engraulis encrasicolus (Linnaeus, 1758) along the Spanish Mediterranean coast. Mar. Pollut. Bull..

[68] Digka N., Tsangaris C., Torre M., Anastasopoulou A., Zeri C. (2018). Microplastics in mussels and fish from the Northern Ionian Sea. Mar. Pollut. Bull..

[69] Duncan E.M., Broderick A.C., Fuller W.J., Galloway T.S., Godfrey M.H., Hamann M., Limpus C.J., Lindeque P.K., Mayes A.G., Omeyer L.C.M. (2019). Microplastic ingestion ubiquitous in marine turtles. Glob. Chang. Biol..

[70] Anastasopoulou A., Kovač Viršek M., Bojanić Varezić D., Digka N., Fortibuoni T., Koren Š., Mandić M., Mytilineou C., Pešić A., Ronchi F. (2018). Assessment on marine litter ingested by fish in the Adriatic and NE Ionian Sea macro-region (Mediterranean). Mar. Pollut. Bull..

[71] Garcia-Garin O., Vighi M., Aguilar A., Tsangaris C., Digka N., Kaberi H., Borrell A. (2019). Boops boops as a bioindicator of microplastic pollution along the Spanish Catalan coast. Mar. Pollut. Bull..

[72] Savoca S., Capillo G., Mancuso M., Faggio C., Panarello G., Crupi R., Bonsignore M., D’Urso L., Compagnini G., Neri F. (2019). Detection of artificial cellulose microfibers in Boops boops from the northern coasts of Sicily (Central Mediterranean). Sci. Total Environ..

[73] Giani D., Baini M., Galli M., Casini S., Fossi M.C. (2019). Microplastics occurrence in edible fish species (Mullus barbatus and Merluccius merluccius) collected in three different geographical sub-areas of the Mediterranean Sea. Mar. Pollut. Bull..

[74] Valente T., Sbrana A., Scacco U., Jacomini C., Bianchi J., Palazzo L., de Lucia G.A., Silvestri C., Matiddi M. (2019). Exploring microplastic ingestion by three deep-water elasmobranch species: A case study from the Tyrrhenian Sea. Environ. Pollut..

[75] Capillo G., Savoca S., Panarello G., Mancuso M., Branca C., Romano V., D’Angelo G., Bottari T., Spanò N. (2020). Quali-quantitative analysis of plastics and synthetic microfibers found in demersal species from Southern Tyrrhenian Sea (Central Mediterranean). Mar. Pollut. Bull..

[76] Capone A., Petrillo M., Misic C. (2020). Ingestion and elimination of anthropogenic fibres and microplastic fragments by the European anchovy (Engraulis encrasicolus) of the NW Mediterranean Sea. Mar. Biol..

[77] Renzi M., Blašković A. (2020). Chemical fingerprint of plastic litter in sediments and holothurians from Croatia: Assessment & relation to different environmental factors. Mar. Pollut. Bull..

[78] Rodríguez-Romeu O., Constenla M., Carrassón M., Campoy-Quiles M., Soler-Membrives A. (2020). Are anthropogenic fibres a real problem for red mullets (Mullus barbatus) from the NW Mediterranean?. Sci. Total Environ..

[79] Savoca S., Bottari T., Fazio E., Bonsignore M., Mancuso M., Luna G.M., Romeo T., D’Urso L., Capillo G., Panarello G. (2020). Plastics occurrence in juveniles of Engraulis encrasicolus and Sardina pilchardus in the Southern Tyrrhenian Sea. Sci. Total Environ..

[80] Carreras-Colom E., Constenla M., Soler-Membrives A., Cartes J.E., Baeza M., Carrassón M. (2020). A closer look at anthropogenic fiber ingestion in Aristeus antennatus in the NW Mediterranean Sea: Differences among years and locations and impact on health condition. Environ. Pollut..

[81] Bayo J., Rojo D., Martínez-Baños P., López-Castellanos J., Olmos S. (2021). Commercial Gilthead Seabream (Sparus aurata L.) from the Mar Menor Coastal Lagoon as Hotspots of Microplastic Accumulation in the Digestive System. Public Health.

[82] Yozukmaz A. (2021). Investigation of microplastics in edible wild mussels from İzmir Bay (Aegean Sea, Western Turkey): A risk assessment for the consumers. Mar. Pollut. Bull..

[83] Sayed A.E.D.H., Hamed M., Badrey A.E.A., Ismail R.F., Osman Y.A.A., Osman A.G.M., Soliman H.A.M. (2021). Microplastic distribution, abundance, and composition in the sediments, water, and fishes of the Red and Mediterranean seas, Egypt. Mar. Pollut. Bull..

[84] Faure F., Saini C., Potter G., Galgani F., de Alencastro L.F., Hagmann P. (2015). An evaluation of surface micro- and mesoplastic pollution in pelagic ecosystems of the Western Mediterranean Sea. Environ. Sci. Pollut. Res..

[85] Suaria G., Avio C.G., Mineo A., Lattin G.L., Magaldi M.G., Belmonte G., Moore C.J., Regoli F., Aliani S. (2016). The Mediterranean Plastic Soup: Synthetic polymers in Mediterranean surface waters. Sci. Rep..

[86] Fastelli P., Blašković A., Bernardi G., Romeo T., Čižmek H., Andaloro F., Russo G.F., Guerranti C., Renzi M. (2016). Plastic litter in sediments from a marine area likely to become protected (Aeolian Archipelago’s islands, Tyrrhenian sea). Mar. Pollut. Bull..

[87] Ruiz-Orejón L.F., Sardá R., Ramis-Pujol J. (2016). Floating plastic debris in the Central and Western Mediterranean Sea. Mar. Environ. Res..

[88] Van der Hal N., Ariel A., Angel D.L. (2017). Exceptionally high abundances of microplastics in the oligotrophic Israeli Mediterranean coastal waters. Mar. Pollut. Bull..

[89] Cannas S., Fastelli P., Guerranti C., Renzi M. (2017). Plastic litter in sediments from the coasts of south Tuscany (Tyrrhenian Sea). Mar. Pollut. Bull..

[90] Mistri M., Infantini V., Scoponi M., Granata T., Moruzzi L., Massara F., De Donati M., Munari C. (2017). Small plastic debris in sediments from the Central Adriatic Sea: Types, occurrence and distribution. Mar. Pollut. Bull..

[91] Abidli S., Antunes J.C., Ferreira J.L., Lahbib Y., Sobral P., Trigui El Menif N. (2018). Microplastics in sediments from the littoral zone of the north Tunisian coast (Mediterranean Sea). Estuar. Coast. Shelf Sci..

[92] Sanchez-Vidal A., Thompson R.C., Canals M., De Haan W.P. (2018). The imprint of microfibres in Southern European deep seas. PLoS ONE.

[93] Simon-Sánchez L., Grelaud M., Garcia-Orellana J., Ziveri P. (2019). River Deltas as hotspots of microplastic accumulation: The case study of the Ebro River (NW Mediterranean). Sci. Total Environ..

[94] Suaria G., Musso M., Achtypi A., Bassotto D., Aliani S. (2020). Textile Fibres in Mediterranean Surface Waters: Abundance and Composition.

[95] Pojar I., Kochleus C., Dierkes G., Ehlers S.M., Reifferscheid G., Stock F. (2021). Quantitative and qualitative evaluation of plastic particles in surface waters of the Western Black Sea. Environ. Pollut..

[96] Bošković N., Joksimović D., Peković M., Perošević-Bajčeta A., Bajt O. (2021). Marine Science and Engineering Microplastics in Surface Sediments along the Montenegrin Coast, Adriatic Sea: Types, Occurrence, and Distribution. J. Mar. Sci. Eng..

[97] Angiolillo M., Gérigny O., Valente T., Fabri M.C., Tambute E., Rouanet E., Claro F., Tunesi L., Vissio A., Daniel B. (2021). Distribution of seafloor litter and its interaction with benthic organisms in deep waters of the Ligurian Sea (Northwestern Mediterranean). Sci. Total Environ..

[98] Miller R.Z., Watts A.J.R., Winslow B.O., Galloway T.S., Barrows A.P.W. (2017). Mountains to the sea: River study of plastic and non-plastic microfiber pollution in the northeast USA. Mar. Pollut. Bull..

[99] Rochman C.M., Tahir A., Williams S.L., Baxa D.V., Lam R., Miller J.T., Teh F.C., Werorilangi S., Teh S.J. (2015). Anthropogenic debris in seafood: Plastic debris and fibers from textiles in fish and bivalves sold for human consumption. Sci. Rep..

[100] Nadal M.A., Alomar C., Deudero S. (2016). High levels of microplastic ingestion by the semipelagic fish bogue Boops boops (L.) around the Balearic Islands. Environ. Pollut..

[101] Naidoo T., Smit A.J., Glassom D. (2016). Plastic ingestion by estuarine mullet Mugil cephalus (Mugilidae) in an urban harbour, KwaZulu-Natal, South Africa. Afr. J. Mar. Sci..

[102] Herrera A., Ŝtindlová A., Martínez I., Rapp J., Romero-Kutzner V., Samper M.D., Montoto T., Aguiar-González B., Packard T., Gómez M. (2019). Microplastic ingestion by Atlantic chub mackerel (Scomber colias) in the Canary Islands coast. Mar. Pollut. Bull..

[103] Le Guen C., Suaria G., Sherley R.B., Ryan P.G., Aliani S., Boehme L., Brierley A.S. (2020). Microplastic study reveals the presence of natural and synthetic fibres in the diet of King Penguins (Aptenodytes patagonicus) foraging from South Georgia. Environ. Int..

[104] Wesch C., Barthel A.K., Braun U., Klein R., Paulus M. (2016). No microplastics in benthic eelpout (Zoarces viviparus): An urgent need for spectroscopic analyses in microplastic detection. Environ. Res..

[105] Comnea-Stancu I.R., Wieland K., Ramer G., Schwaighofer A., Lendl B. (2017). On the Identification of Rayon/Viscose as a Major Fraction of Microplastics in the Marine Environment: Discrimination between Natural and Manmade Cellulosic Fibers Using Fourier Transform Infrared Spectroscopy. Appl. Spectrosc..

[106] Faruk O., Bledzki A.K., Fink H.P., Sain M. (2014). Progress report on natural fiber reinforced composites. Macromol. Mater. Eng..

[107] Röder T., Moosbauer J., Wöss K., Schlader S., Kraft G. (2013). Man-Made Cellulose Fibres-a Comparison Based on Morphology and Mechanical Properties. Lenzinger Berichte.

[108] Bredereck K., Hermanutz F. (2008). Man-made cellulosics. Color. Technol..

[109] Morseletto P. (2020). A new framework for policy evaluation: Targets, marine litter, Italy and the Marine Strategy Framework Directive. Mar. Policy.

[110] Henry B., Laitala K., Klepp I.G. (2019). Microfibres from apparel and home textiles: Prospects for including microplastics in environmental sustainability assessment. Sci. Total Environ..

[111] Stagioni M., Montanini S., Vallisneri M. (2011). Feeding habits of European hake, Merluccius merluccius (Actinopterygii: Gadiformes: Merlucciidae), from the northeastern Mediterranean sea. Acta Ichthyol. Piscat..

[112] Matić-Skoko S., Šegvić-Bubić T., Mandić I., Izquierdo-Gomez D., Arneri E., Carbonara P., Grati F., Ikica Z., Kolitari J., Milone N. (2018). Evidence of subtle genetic structure in the sympatric species Mullus barbatus and Mullus surmuletus (Linnaeus, 1758) in the Mediterranean Sea. Sci. Rep..

[113] Anastasopoulou A., Mytilineou C., Smith C.J., Papadopoulou K.N. (2013). Plastic debris ingested by deep-water fish of the Ionian Sea (Eastern Mediterranean). Deep. Res. Part I Oceanogr. Res. Pap..

[114] Matiddi M., Hochsheid S., Camedda A., Baini M., Cocumelli C., Serena F., Tomassetti P., Travaglini A., Marra S., Campani T. (2017). Loggerhead sea turtles (Caretta caretta): A target species for monitoring litter ingested by marine organisms in the Mediterranean Sea. Environ. Pollut..

[115] Boerger C.M., Lattin G.L., Moore S.L., Moore C.J. (2010). Plastic ingestion by planktivorous fishes in the North Pacific Central Gyre. Mar. Pollut. Bull..

[116] Cole M., Webb H., Lindeque P.K., Fileman E.S., Halsband C., Galloway T.S. (2014). Isolation of microplastics in biota-rich seawater samples and marine organisms. Sci. Rep..

[117] Gwinnett C., Miller R.Z. (2021). Are we contaminating our samples? A preliminary study to investigate procedural contamination during field sampling and processing for microplastic and anthropogenic microparticles. Mar. Pollut. Bull..

[118] Ng K.L., Obbard J.P. (2006). Prevalence of microplastics in Singapore’s coastal marine environment. Mar. Pollut. Bull..

[119] Prata J.C., Reis V., da Costa J.P., Mouneyrac C., Duarte A.C., Rocha-Santos T. (2021). Contamination issues as a challenge in quality control and quality assurance in microplastics analytics. J. Hazard. Mater..

[120] Cowger W., Booth A.M., Hamilton B.M., Thaysen C., Primpke S., Munno K., Lusher A.L., Dehaut A., Vaz V.P., Liboiron M. (2020). Special Issue: Microplastics Reporting Guidelines to Increase the Reproducibility and Comparability of Research on Microplastics. Appl. Spectrosc..

[121] Miller E., Sedlak M., Lin D., Box C., Holleman C., Rochman C.M., Sutton R. (2021). Recommended best practices for collecting, analyzing, and reporting microplastics in environmental media: Lessons learned from comprehensive monitoring of San Francisco Bay. J. Hazard. Mater..

[122] Gaylarde C., Baptista-Neto J.A., da Fonseca E.M. (2021). Plastic microfibre pollution: How important is clothes’ laundering?. Heliyon.

[123] Zayen A., Sayadi S., Chevalier C., Boukthir M., Ben Ismail S., Tedetti M. (2020). Microplastics in surface waters of the Gulf of Gabes, southern Mediterranean Sea: Distribution, composition and influence of hydrodynamics. Estuar. Coast. Shelf Sci..

[124] Schönlau C., Karlsson T.M., Rotander A., Nilsson H., Engwall M., van Bavel B., Kärrman A. (2020). Microplastics in sea-surface waters surrounding Sweden sampled by manta trawl and in-situ pump. Mar. Pollut. Bull..

[125] Kuklinski P., Wicikowski L., Koper M., Grala T., Leniec-Koper H., Barasiński M., Talar M., Kamiński I., Kibart R., Małecki W. (2019). Offshore surface waters of Antarctica are free of microplastics, as revealed by a circum-Antarctic study. Mar. Pollut. Bull..

[126] Scopetani C., Esterhuizen-Londt M., Chelazzi D., Cincinelli A., Setälä H., Pflugmacher S. (2020). Self-contamination from clothing in microplastics research. Ecotoxicol. Environ. Saf..

[127] Grigorakis S., Mason S.A., Drouillard K.G. (2017). Determination of the gut retention of plastic microbeads and microfibers in goldfish (Carassius auratus). Chemosphere.

